# Digging Signatures in 13-Month-Old 3xTg-AD Mice for Alzheimer's Disease and Its Disruption by Isolation Despite Social Life Since They Were Born

**DOI:** 10.3389/fnbeh.2020.611384

**Published:** 2021-01-18

**Authors:** Lydia Gimenez-Llort, Daniel Alveal-Mellado

**Affiliations:** ^1^Institut de Neurociències, Universitat Autònoma de Barcelona, Barcelona, Spain; ^2^Department of Psychiatry and Forensic Medicine, School of Medicine, Universitat Autònoma de Barcelona, Barcelona, Spain

**Keywords:** OCD, 3xTg-AD, BPSD, Translational neuroscience, Neurospsychiatric symptoms, Loneliness, isolation, COVID-19

## Abstract

The severity of this pandemic's scenarios will leave significant psychological traces in low resistant and resilient individuals. Increased incidence of depression, anxiety, obsessive-compulsive disorder (OCD), and post-traumatic stress disorder has already been reported. The loss of human lives and the implementation of physical distance measures in the pandemic and post-COVID scenarios may have a greater impact on the elderly, mostly in those with dementia, as OCD and other neuropsychiatric symptoms (NPS) are quite prevalent in this population. Modeling NPS in animals relies in neuroethological perspectives since the response to new situations and traumatic events, critical for survival and adaptation to the environment, is strongly preserved in the phylogeny. In the laboratory, mice dig vigorously in deep bedding to bury food pellets or small objects they may find. This behavior, initially used to screen anxiolytic activity, was later proposed to model better meaningless repetitive and perseverative behaviors characteristic of OCD or autism spectrum disorders. Other authors found that digging can also be understood as part of the expression of the animals' general activity. In the present brief report, we studied the digging ethograms in 13-month-old non-transgenic and 3xTg-AD mice modeling normal aging and advanced Alzheimer's disease (AD), respectively. This genetic model presents AD-like cognitive dysfunction and NPS-like phenotype, with high mortality rates at this age, mostly in males. This allowed us to observe the digging pattern's disruption in a subgroup of 3xTg-AD mice that survived to their cage mates. Two digging paradigms involving different anxiogenic and contextual situations were used to investigate their behavior. The temporal course and intensity of digging were found to increase in those 3xTg-AD mice that had lost their “room partners” despite having lived in social structures since they were born. However, when tested under neophobia conditions, this behavior's incidence was low (delayed), and the temporal pattern was disrupted, suggesting worsening of this NPS-like profile. The outcomes showed that this combined behavioral paradigm unveiled distinct features of digging signatures that can be useful to study these perseverative behaviors and their interplay with anxiety states already present in the AD scenario and their worsening by naturalistic/forced isolation.

**Graphical Abstract:**
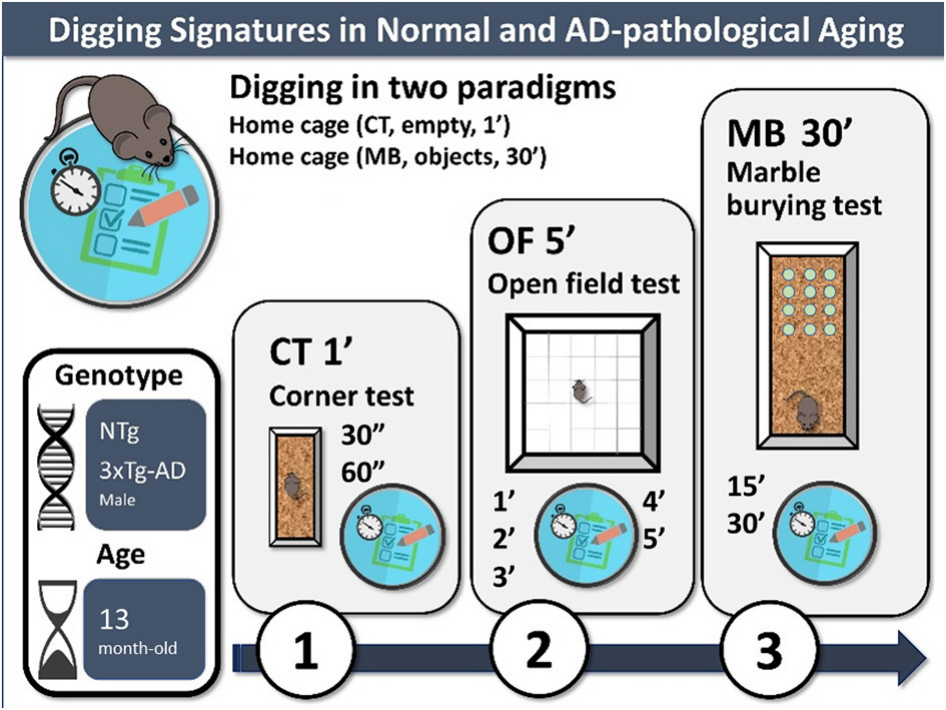
Digging signatures in normal and AD-pathological aging. Experimental design: 3-days battery of tests to assess digging in two home cage paradigms (CT, corner test, 1 min; MB, marble burying test with 12 marbles), also including an open-field test to control for exploration, anxiety, and emotionality. In the present work, 13-month-old NTg (normal aging) and 3xTg-AD mice (advanced stages of disease) were assessed.

## Introduction

On the day that the COVID-19 pandemic took one million people's life, there is no doubt that the severity of the COVID-19 pandemic will leave important psychological traces in low resistant and/or resilient individuals (Farhan and Llopis, 2020). The first clinical reports already reported increased incidence of sleep disorders, depression, anxiety, as well as obsessive-compulsive disorder (OCD), and post-traumatic stress disorder (Banerjee, 2020; Rivera and Carballea, 2020). Since then, the number of reports on OCD, a complex disorder due to its diagnostic classification and clinical heterogeneity (Ruscio et al., 2010), has fast increased (Kumar and Somani, 2020; Shafran et al., 2020; Sulaimani and Bagadood, 2020). The American Psychiatry Association^1^ defines this mental health disorder as a series of recurring unwanted and distressing thoughts, ideas or sensations (obsessions) and compulsive repetitive behaviors (such as hand washing, cleaning) or mental acts aimed to decrease the resulting distress or according to rules. Pre-pandemic descriptive epidemiology of OCD referred to a prevalence of 3.1% of the general population and the association of this disorder with significant interference of social interactions, daily activities, and reduced quality of life (Fontenelle et al., 2006; Ruscio et al., 2010). In the current situation, the clinical relevance of OCD has increased, and the consequent worsening of daily life (Fontenelle and Miguel, 2020) has urged the elaboration of guides with international consensus for the management of OCD during COVID-19 and post-COVID-19 scenarios (Fineberg et al., 2020).

Among older people, the impact of COVID-19 is devastating (Van Loon et al., 2020), mainly among the frailest living in long-term care homes (Commas-Herrera et al., 2020). In these nursery care settings, most residents are affected by dementia (Seitz et al., 2010), a population that already has higher mortality rates than aged-matched control populations, with males showing deranged neuro-immuno-endocrine system and worse survival than females despite not as bad neuropathological status (van Dijk et al., 1991; Mitchell et al., 2010). On the other hand, despite the current social and working roles can make female workers more exposed to COVID-19, the first study on gender differences in COVID-19 found both genders having the same prevalence of the disease, but males being more at risk for worse outcomes and death independently of the age (Jin et al., 2020). Dementia care during COVID-19 is a challenge (Wang et al., 2020). In most home-care centers, the confinement and implementation of physical distance measures, with patients being segregated and relocated in new rooms to protect them from the virus, is resulting in social isolation. It is well-known that social vulnerability, frailty, and mortality in older adults is a worrisome triad (Andrew et al., 2008). Social isolation increases the risk of dementia (Wilson et al., 2007) and enhances not only its hallmark cognitive decline but neuropsychiatric disorders, which are already quite prevalent in these patients (Cummings, 1997; Seitz et al., 2010; Zhao et al., 2016). The management of their neuropsychiatric symptoms (NPS) is considered a major issue for the patients' and caregivers' quality of life (Kamiya et al., 2014).

At the translational level, modeling NPS, also known as behavioral and psychological symptoms associated with dementia (BPSD), in rodents relies in neuroethological approaches since the response to different kind of situations and traumatic events, critical for survival and adaptation to the environment, are strongly preserved in the phylogeny (Giménez-Llort et al., 2007). In the wild, burying was first described as a defensive response in rats and considered as reflecting the anxiety state of animals (Pinel and Treit, 1978). In the laboratory, mice dig vigorously in deep bedding to bury food pellets or small objects such as marbles (Gyertyan, 1995). Thus, digging and marble burying have been proposed as simple methods for *in vivo* identification of biological impacts in mice (Broekkamp et al., 1986; Deacon, 2006). This behavior, initially used to screen anxiolytic activity, was later proposed to model meaningless repetitive and perseverative behaviors as compulsive-like characteristics of OCD or autism spectrum disorders in rodents (de Brouwer et al., 2020; Mahmood et al., 2020). Also, the animal's general activity can be a confounding factor and, conversely, digging can also be understood as a measure of general activity rather than a measure of repetitive or anxiety-related behavior (Greene-Schloesser et al., 2011; Mitra et al., 2017; de Brouwer et al., 2019; Dixit et al., 2020). The controversy around the digging behavior and what the paradigm might measure is open.

Spontaneous (non-induced) compulsive-like mice based on bidirectional selection for excessive, repetitive, and perseverant digging behaviors are considered mouse models of OCD exhibiting good face, predictive, and construct validity (Greene-Schloesser et al., 2011; Mitra et al., 2017). In our precedent studies with the 3xTg-AD mice, a genetic model of Alzheimer's disease (Oddo et al., 2003) that presents AD-cognitive dysfunction but also a conspicuous BPSD-like phenotype (Giménez-Llort et al., 2006, 2007), we showed increased marble-burying in middle-aged (12-month-old) male 3xTg-AD mice as compared to age-matched non-transgenic (NTg) mice with normal aging (Torres-Lista et al., 2015). Their marble-burying response was related to neophobia and shown to be modulated by repeated handling, reversed by chronic treatment with neuroleptic risperidone (Torres-Lista et al., 2015), and modified by chronic caffeine (Baeta-Corral et al., 2018). In a social paradigm, elicitation of digging behavior was similar among males independently of their genotype but early expressed in female 3xTg-AD mice (Torres-Lista and Giménez-Llort, 2019). In our most recent work, in male 3xTg-AD mice under a long-term isolation (Muntsant and Giménez-Llort, 2020a) increased gross and fine motor activity was recorded, with enhanced nest-building. This is also a species-specific natural behavior that, when disrupted, some authors consider a homologous to hoarding in humans with OCD (Warneke, 1993) and modeling compulsive-like behavior in mice (Greene-Schloesser et al., 2011). Recently, during housing routines, we observed that the spontaneous digging behavior elicited when an animal is transferred to a new home cage with clean beddings was disrupted in a subgroup of old male 3xTg-AD mice that had recently lost their cage-mates. This observation was restricted to males and AD-genotype since the increased mortality rates of male 3xTg-AD mice (Giménez-Llort et al., 2008) often lead to some males living alone for such natural reasons.

Given the above studies in 3xTg-AD mice and the current clinical reports of increased incidence of OCD and anxiety disorders (Banerjee, 2020; Rivera and Carballea, 2020) due to strict physical distance measures, a brief translational study was designed. The work aimed to confirm the observations of derangement of digging patterns in the subgroup of male 3xTg-AD mice that, after 10 months of social life, lost their partners and lived alone during the last 2–3 months. We aimed to define the nuances of their digging patterns and their expression under different anxiogenic conditions, as they are also serving as a behavioral tool for current investigations. Therefore, digging patterns were assessed in 13-months-old NTg and the two subgroups of 3xTg-AD male mice, an age that corresponds to normal aging and advanced stages of the disease, respectively (Belfiore et al., 2019). The expression of digging behavior was recorded in experimental scenarios where a home-cage with beddings is used, such as the corner test (change to a new and clean cage) and the marble tests, also involving different anxiogenic and contextual situations (home-cage with beddings, and without/with objects, respectively). The open-field test was included to monitor the animal's general activity in a classical anxiogenic environment without beddings.

## Materials and Methods

### Animals

A total number of 43 13-month-old male homozygous 3xTg-AD (*n* = 28) and non-transgenic (NTg, *n* = 15) mice on a C57BL/6J background (after embryonic transfer and backcrossing at least 10 generations) established in the Universitat Autònoma de Barcelona (Baeta-Corral and Giménez-Llort, 2014) were used in this study. The 3xTg-AD mice harboring transgenes were genetically engineered at the University of California Irvine, as previously described (Oddo et al., 2003). Animals were maintained in groups of 3–4 mice per cage (Macrolon, 35 × 35 × 25 cm) filled with 5 cm of clean wood cuttings (Ecopure, Chips6, DateSand, UK; Uniform cross-cut wood granules with 2.8–1.0 mm chip size) and nesting materials (Kleenex, Art: 08834060, 21 × 20 cm, White). In the current work, seven of the 28 3xTg-AD mice had lost their cage-mates and lived alone in their cage for 2–3 months. In all the cases, the standard home cages covered with a metallic grid allow the perception of olfactory and auditory stimuli from the rest of the colony. All the animals were maintained under standard laboratory conditions of food and water ad lib, 22 ± 2°C, 12 h light: dark cycle with lights on at 8:00 am, and relative humidity 50–60%.

### Behavioral Assessment

Digging and other behaviors were measured in the corner test, open-field test, and marble burying test under dim white light (20 lx) during their light phase of the light: dark cycle (from 10 am to 1 pm) (see Graphical abstract).

Behavioral assessments were performed by direct observation by two independent observers blind to the genotype, in a counterbalanced manner and with the support of a computerized video system. All procedures followed the Spanish legislation on the “Protection of Animals Used for Experimental and Other Scientific Purposes” and the EU Directive (2010/63/UE) on this subject. The study complies with the ARRIVE guidelines developed by the NC3Rs and aims to reduce the number of animals used (Kilkenny et al., 2010).

Day 1. Digging in a new home cage only with clean wood cuttings was assessed in a corner test (CT). The animal was introduced in the cage's center, and the neophobia response was observed for 1 min. The number of visited corners before the elicitation of the first rearing (CTcbR) and the latency of rearing (CTlatR) were noted. Then, the number of visited corners (CTc) and rearings (CTr) were recorded at 30 and 60 s. The ratio of these variables (Ratio CTc/r) was calculated. The number of digging episodes (>2 s, in order to avoid false-positive observations) elicited during 30 and 60 s were noted. Prevalence of digging was recorded at the end of the test.

Day 3. The animals were placed in the center of an open-field (metalwork, beige, 44 × 38 × 10 cm height), and their general and vertical activity (R, rearings) was recorded for 5 min. The time and distance covered in the center and periphery during the test were recorded using the VideoTrack analysis system (ViewPoint Behavior Technology, Lyon, France) (Giménez-Llort et al., 1995). Walking speed was calculated as the distance covered per unit of time. In those animals where distance and time in the periphery were zero, zero was given to the walking speed. The number of defecations boli (Def) and presence/absence of urination (Ur) were also noted. Prevalence of rearing, defecation boli, and urination were recorded at the end of the test.

Day 12. One week later, animals were assessed in the marble-burying test (MB). The animal was introduced facing the wall of a home-cage. Twelve glass marbles (1.5 cm diameter, 8 g) were evenly spaced (four rows in the cage's upper area) on a 5 cm thick layer of clean wood cuttings. The mice were left in the cage with marbles for a 30 min period. The evaluation was done twice to record the temporal course at 15 and 30 min. (Santana-Santana and Giménez-Llort, Submitted). Digging in the new home cage was measured by the level of marbles' burying: “Buried” (B, 100% buried), those left “Intact” (I), and the remaining subset considered as those “Moved or partially buried” (M).

### Statistics

Results are expressed as mean ± SEM. SPSS 20.0 software was used. One way ANOVA followed by *post-hoc* Duncan's test was used to compare the three independent groups. Differences within the same group were analyzed with a Paired *t*-test. For categorical variables, Fisher's exact test with 2 × 2 was used. In all the tests, *p* < 0.05 was considered statistically significant.

## Results

As a first step, we investigated the genotype differences, those due to the three human transgenes. Table 1 summarizes the results of the 30 variables studied and point at vertical exploratory activity as the most sensitive variable to show genotype differences, with a severe drop of rearing behavior in the periphery. Also, a reduced number of moved marbles in favor of an increase in those buried lead to genotype differences.

**Table 1:** Genotype differences between 13-month-old male 3xTg-AD mice and NTg mice in the corner, open-field, and marble tests.

	**NTg mice *n* = 15 (Mean ± SEM)**	**3xTg-AD mice *n* = 28 (Mean ± SEM)**	**Genotype differences**
**1.Corner test**			
CTcbR (*n*)	4.64 ± 0.58	4.00 ± 0.53	*n.s*.
CTlatR (*n*)	13.40 ± 1.90	11.64 ± 1.23	*n.s*.
CTc30 (*n*)	9.93 ± 0.71	9.36 ± 0.89	*n.s*.
CTr30 (*n*)	4.33 ± 0.42	3.64 ± 0.44	*n.s*.
Ratio CTc/r30 (index)	2.74 ± 0.55	2.95 ± 0.24	*n.s*.
CTc60 (*n*)	15.13 ± 0.89	13.14 ± 1.18	*n.s*.
CTr60 (*n*)	9.60 ± 0.86	5.93 ± 0.66	**
Ratio CTc/r60 (index)	1.77 ± 0.19	2.66 ± 0.26	**
Digging 30” (*n* of episodes)	0.07 ± 0.07	0.25 ± 0.11	*n.s*.
Digging 60” (*n* of episodes)	0.80 ± 0.24	0.89 ± 0.26	*n.s*.
**2. Open field test**			
Distance covered in the center (cm)	86.49 ± 10.59	72.07 ± 6.09	*n.s*.
Distance covered in the periphery (cm)	266.57 ± 10.99	78.13 ± 9.17	***
Time spent in the center (s)	15.43 ± 2.31	27.98 ± 1.84	***
Time spent in the periphery (s)	49.84 ± 1.14	16.70 ± 1.76	***
Walking speed in the center (cm/s)	8.81 ± 0.71	3.07 ± 0.23	***
Walking speed in the periphery (cm/s)	5.50 ± 0.23	3.02 ± 0.42	***
R1 (*n*)	3.20 ± 0.83	0.04 ± 0.01	***
R2 (*n*)	4.20 ± 1.08	0.43 ± 0.08	**
R3 (*n*)	4.07 ± 1.05	0.46 ± 0.09	***
R4 (*n*)	5.00 ± 1.29	0.93 ± 0.18	***
R5 (*n*)	3.80 ± 0.98	1.00 ± 0.19	***
Total rearings (*n*)	20.27 ± 5.23	2.86 ± 0.54	***
Defecation (*n* of boli)	0.33 ± 0.09	4.11 ± 0.78	***
Urination (presence/absence)	0.00	0.74 ± 0.14	***
**3. Marble test**			
I15 (*n*)	7.20 ± 0.87	6.64 ± 0.73	*n.s*.
M15 (*n*)	1.33 ± 0.43	1.07 ± 0.37	*n.s*.
B15 (*n*)	3.47 ± 0.83	4.21 ± 0.67	*n.s*.
I30 (*n*)	2.80 ± 0.63	3.64 ± 0.71	*n.s*.
M30 (*n*)	2.20 ± 0.61	0.75 ± 0.23	*
B30 (*n*)	7.00 ± 0.74	7.64 ± 0.75	*n.s*.
Student's *t-*test, ***p < 0.001, **p < 0.01, *p < 0.05, n.s. *p* > 0.05 vs. NTg mice.			

*Corner test: CTcbR, visited corners before the first rearing; CTlatR, latency of rearings in seconds; CTcn, visited corners at n s; CTrn, rearings at n s; Ratio CTc/r n, ratio visited corners divided by rearings at n s of the test; Digging in the corner test at 30 (D30) and 60 (D60) s. Open-field test: Rn, rearings at minute n; Marble test: marbles left intact (I15, I30), half-buried or moved (M15, M30) or completely buried (B15, B30) at 15 and 30 min, respectively*.

**Figure 1:**
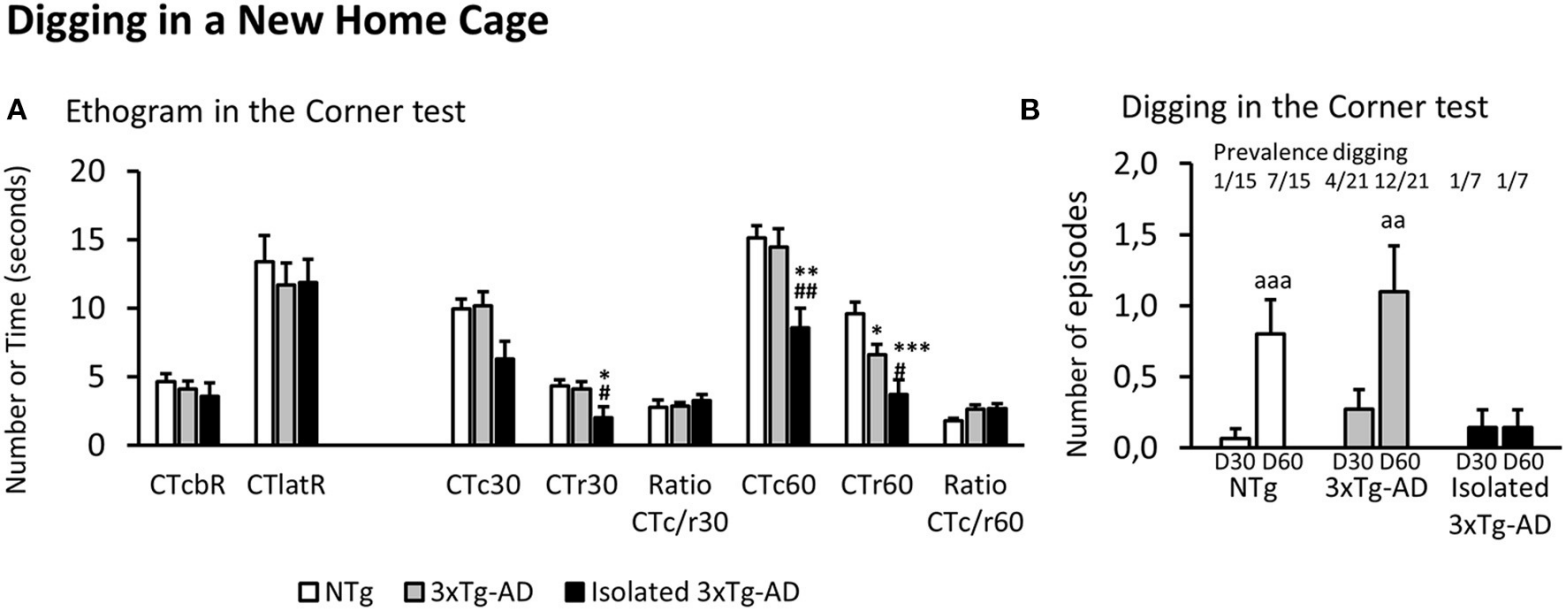
Ethogram and Digging in a new home cage (Corner test). Results are expressed as mean ± SEM. **(A)** Ethogram in the corner test: CTcbR, visited corners before the first rearing; CTlatR, latency of rearings in seconds; CTc*n*, visited corners at *n* s; CTr*n*, rearings at *n* s; Ratio CTc/r n, ratio visited corners divided by rearings at *n* s of the test. **(B)** Digging in the corner test at 30 (D30) and 60 (D60) s. Statistics: One-way ANOVA followed by *post-hoc* Duncan's test, *p < 0.05, **p < 0.01, and ***p < 0.001 vs. the NTg group; #*p* < 0.05 and ##*p* < 0.01 vs. the 3xTg-AD mice. Paired *t*-test, aa *p* < 0.01 and aaa *p* < 0.001 vs. the same group at 30 s.

**Figure 2:**
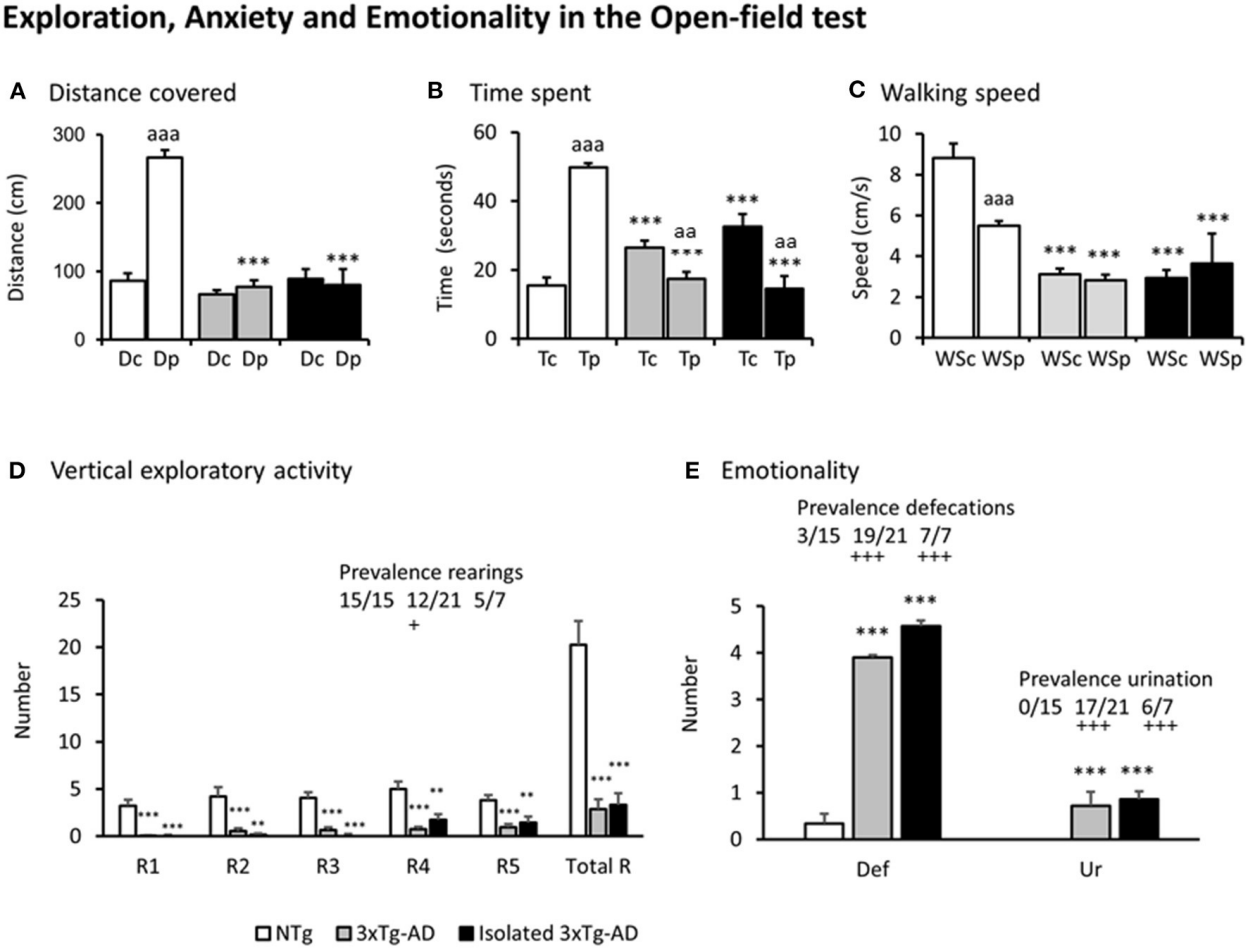
Exploration, Anxiety, and Emotionality in the Open-field test. Results are expressed as mean ± SEM. **(A)** Distance covered, **(B)** Time spent, **(C)** Walking speed, **(D)** Vertical exploratory activity, **(E)** Emotionality. Abbreviations: Dc, distance in the center; Dp, distance in the periphery; Tc, time in the center; Tp, time in the periphery; WSc, Walking speed in the center; WSp, walking speed in the periphery; Rn, rearings at minute n; Total R, total number of rearings; Def, defecation boli; Ur, urination. Statistics: One-way ANOVA followed by *post-hoc* Duncan's test, *p < 0.05, **p < 0.01, ***p < 0.001 vs. the NTg mice; #*p* < 0.05. vs. the 3xTg-AD mice, Paired *t*-test, aa *p* < 0.01, aaa *p* < 0.001 vs. the same variable in the center of the open-field test. Fisher's exact test, +*p* < 0.05 and +++*p* < 0.001 vs. the NTg mice.

**Figure 3:**
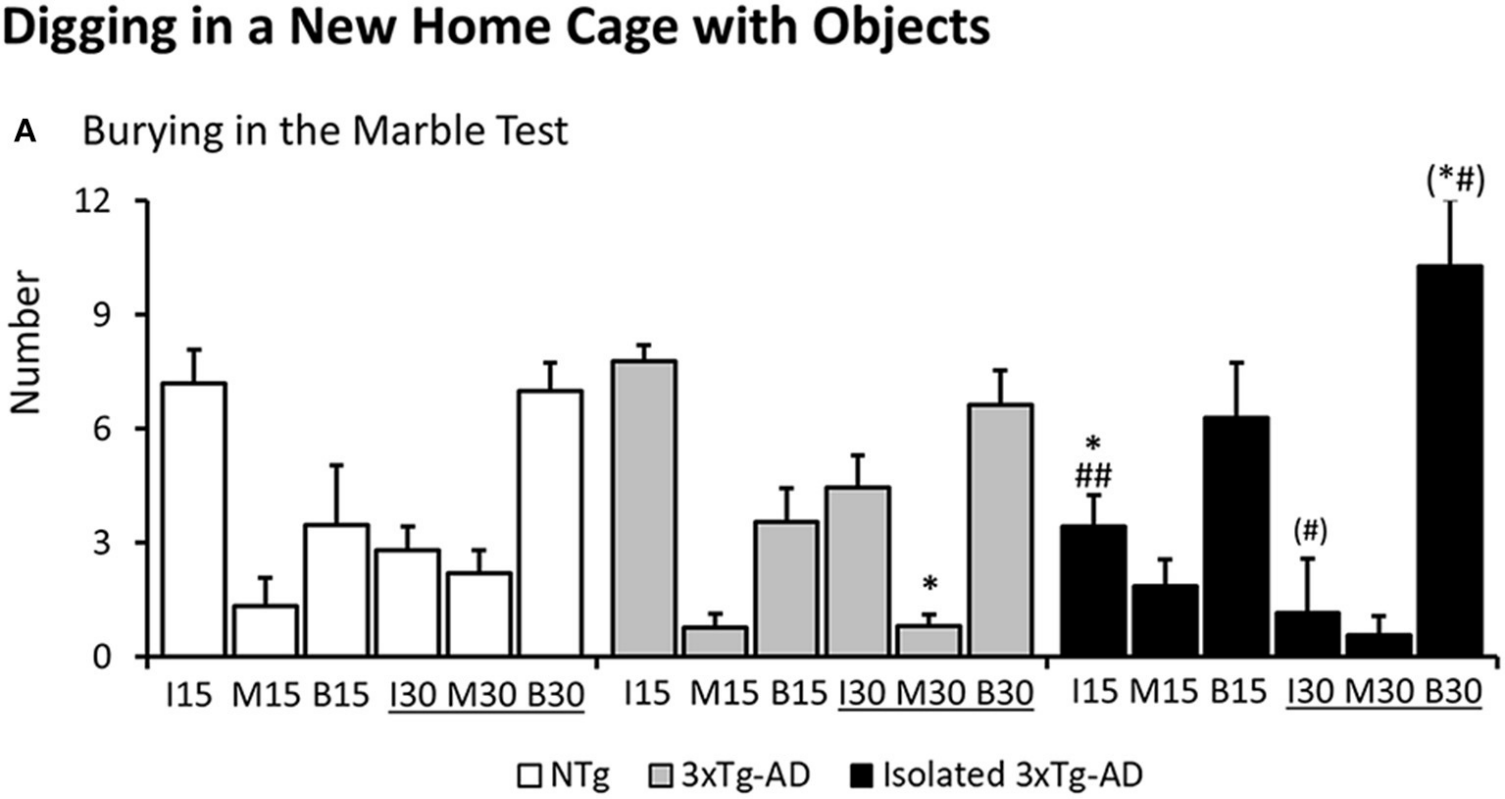
Digging in a new home cage with small objects (Marble test). Results are expressed as mean ± SEM **(A)** At 15 and 30 min of the test, the number of marbles left intact (I15, I30), half-buried or moved (M15, M30) or completely buried (B15, B30) is indicated. Statistics: One-way ANOVA followed by *post-hoc* Duncan's test, *p < 0.05, **p < 0.01, and ***p < 0.001 vs. the NTg mice; #*p* < 0.05. vs. the 3xTg-AD mice.

To verify our hypothesis that the 3xTg-AD mice that recently lost their home-cage partners exhibited different digging patterns, the data of 3xTg-AD mice was depicted in two subgroups, according to their most recent housing conditions. The results clearly showed that while NTg (*n* = 15) and 3xTg-AD mice (*n* = 21) were quite alike in most behaviors, genotype differences emerged in the subset of 3xTg-AD mice (*n* = 7) that recently lost their partners. The following paragraphs, illustrated in Figures 1–3, show the nuances in the digging patterns elicited in two distinct home-cage settings with beddings.

As illustrated in Figure 1A, in all the groups, the ethogram of neophobia in the corner test started with an inspection of the cage (*n* = 43, 4.18 ± 0.39 visited corners) followed by a first rearing (*n* = 43, latency: 12.30 ± 1.02 s). At 30 s, NTg and 3xTg-AD mice permanently living in social conditions showed similar values in the total number of visited corners and rearings. However, the behavior of isolated 3xTg-AD mice was significantly reduced to 62% of corners and 48% of rearings, reaching statistical significance the drop in vertical activity [*F*(2, 40) = 3.765, *p* = 0.0318; *post-hoc, p* < 0.05 vs. each of the other two groups]. The ratio between horizontal and vertical activity was maintained equal in all the groups (*n* = 43, 2.87 ± 0.24). At the end of the test (60 s), the drop of vertical activity [*F*(2, 40) = 8.034, *p* = 0.0017] was now observable in both subgroups of 3xTg-AD compared to NTg mice. However, it was more intense in the isolated 3xTg-AD mice (*post-hoc, p* < 0.05, 3xTg-AD vs. NTg; but *p* < 0.001, isolated 3xTg-AD vs. NTg) that also differed from those permanently living in social groups (*p* < 0.05, isolated 3xTg-AD vs. 3xTg-AD). The horizontal activity also showed a group effect [*F*(2, 40) = 4.565, *p* = 0.0164], but it was due to a drop of total visited corners in isolated 3xTg-AD mice (*p* < 0.01, isolated 3xTg-AD vs. the other two groups). The ratio CTc/r of NTg dropped to 64% while that of both subgroups of 3xTg-AD mice was maintained (*n* = 28, 2.63 ± 0.26), albeit these differences did not reach statistical significance.

Digging behavior at 30 and 60 s in the corner test is illustrated in Figure 1B. Prevalence of digging behavior in the new home cage at 30 s was scarce, as it was only shown by 1/15 (6.7%) NTg mice, 4/21(19%) 3xTg-AD mice, and 1/7 (14%) in the subgroup of isolated 3xTg-AD mice. Thereafter, the behavior increased in NTg (prevalence 7/15, 47%; incidence 6/15 40%) and 3xTg-AD (prevalence 12/21, 57%; incidence 8/21, 33%) mice. In both groups, the ethogram measured as the number of digging episodes showed scarce emergence during the first 30 s and increased at 60 s (accumulated counts). This temporal pattern was statistically significant in both groups (NTg mice, D60 vs. D30, *p* < 0.001; 3xTg-AD mice, D60 vs. D30, *p* < 0.01). In contrast, no new digging behavior episodes were observable among isolated 3xTg-AD mice.

Figures 2A–E, illustrate the different variables assessed in the open-field test. Except for the distance covered in the central area of the field where all the groups covered a similarly short distance [*F*(2, 212) = 1.894, *p* = 0.153], all the other variables showed that the pattern of NTg mice was distinct from that of the two subgroups of 3xTg-AD mice [all *F*'s(2, 212) > 9.327, *p* < 0.000131]. In particular, thigmotaxis was different among groups as shown by preference (in time spent, *p* = 1.16 e-21, and distance covered *p* = 1.89 e-19) for the periphery shown by NTg mice, but lost in both groups of 3xTg-AD mice for distance and inverted in terms of time (3xTg-AD mice, *p* = 0.00628; isolated 3xTg-AD mice, *p* = 0.00214) (Figures 2A,B). This was due to increased freezing behaviors in the center. The total distance covered by both subgroups of 3xTg-AD mice in the periphery was as scarce as in the center and drastically lower than in NTg mice. The distance and time spent allowed to calculate the animals' averaged walking speed (Figure 2C). The walking speed was different among groups since both 3xTg-AD groups walked slower than NTg mice and independently of the open-field area. The paired analysis indicated that NTg mice walked faster in the center than in the periphery (*p* < 0.001). This was confirmed by their sustained rearing behavior levels (Figure 2D) through the 5 min of the test. Only 12/21 (57%) group-housed 3xTg-AD and 5/7 (71%) isolated 3xTg-AD mice performed rearings, and their number was small. Statistically significant differences in the prevalence were shown between 3xTg-AD vs. NTg mice (*p* < 0.05).

Emotionality, as measured by defecation and urination, was found increased in both subgroups of 3xTg-AD mice as compared to NTg mice [both *F*'s(2, 40) > 16.4, *p* < 6.78 e-06; *post-hoc p* < 0.001 vs. NTg]. Also, 19/21 (90%) grouped and 7/7 (100%) isolated 3xTg-AD defecated during the test, while only 3/15 (20%) NTg mice did so (both, *p* < 0.001 vs. to NTg mice). The presence of urination was only shown in 3xTg-AD mice (*n* = 24, 0.72 ± 0.09, absent in NTg, *p* < 0.001), with a prevalence of 17/21 (81%) in group-housed 3xTg-AD mice and 6/7 (86%) in those isolated (both, *p* < 0.001 vs. NTg mice).

As illustrated in Figure 3, digging in the marble test was different among groups as measured by the number of marbles left intact at 15 min [*F*(2, 40) = 4.283 *p* = 0.0206]. NTg and 3xTg-AD mice showed similar patterns. However, digging behavior in the subgroup of isolated 3xTg-AD mice was increased as measured by a reduced number of marbles left intact (*post-hoc* isolated 3xTg-AD vs. NTg *p* = 0.0202; vs. 3xTg-AD *p* = 0.0086). At the end of the test, differences in the number of marbles half-buried o moved were found among groups [*F*(2, 40) = 3.551, *p* = 0.0381, *post-hoc*, 3xTg-AD mice vs. NTg *p* = 0.0211]. *Post-hoc* analysis also detected differences between isolated 3xTg-AD mice and 3xTg-AD in the number of marbles left intact at the end of the test [*F*(2, 40) = 3.180, *p* = 0.0523, but *post-hoc*, isolated 3xTg-AD mice vs. 3xTg-AD *p* = 0.0284] and buried [*F*(2, 40) = 2.938, *p* = 0.0645, but *post-hoc*, isolated 3xTg-AD mice vs. NTg *p* = 0.0429 and vs. 3xTg-AD *p* = 0.0305].

## Discussion

Species-typical behaviors can provide neuroethological tools to follow up the animal's well-being, age/aging and disease processes, and modulation by treatments and environmental factors (Oddo et al., 2003; Greene-Schloesser et al., 2011). Digging can be spontaneously elicited when animals are transferred into a new home-cage with clean beddings during housing routines but can also be reproduced in experimental scenarios such as the corner and the marble tests. In the present brief report, two digging paradigms involving different anxiogenic and contextual situations were used to investigate the digging patterns in the 13-month-old males with normal and genetically induced AD-pathological aging. We confirmed the disruptive effects of short isolation (2–3 months) observed in a subgroup of 3xTg-AD that recently lost their cage-mates after 10 months living in a standard social environment. These 3xTg-AD mice that survived to their partners indicated that their current social housing conditions modified the patterns, achieving divergence with NTg mice but also 3xTg-AD mice still living in social groups.

Elicitation of digging behavior can be more or less conspicuous, depending on many intrinsic and extrinsic factors, and it is usually under-recorded. Slight methodological adaptations of the corner test and the marble test protocols were made to enhance the chances to record digging behavior. The changes were related to the windows and frames of observation. The corner test, performed at 30 s, was prolonged until 60 s. In the marble test, measures were taken at 15 min, and the end of the test to record the temporal progression (Santana-Santana and Giménez-Llort, Submitted).

The results show the consistency of some behavioral patterns, independently of the genotype or social condition. However, the short battery of tests also allowed to confirm the emergence of disruptions in digging in the 3xTg-AD mice and, among them, in those that lost their cage-mates. This “natural isolation” subgroup performed worse than expected. As described in the present work, for the first time, the analysis of the segregated data unveiled distinct modulation of digging signatures in terms of prevalence, intensity, and patterns, depending on the context. When tested for neophobia (immediate fear of a new place), the prevalence was lower, and the digging pattern was mostly absent. However, the activity patterns, thigmotaxis, and emotionality of both 3xTg-AD mice subgroups did not differ in the open-field test. In this classical test for anxiety, the phenotype of all the 3xTg-AD mice was different from that shown by NTg mice with normal aging. According to the action program by Lát (1973), describing the immediate fearful response and the subsequent actions developed by the animal confronted to a new environment, the open-field elicited a severe anxiety/fear-like pattern in both subgroups of 3xTg-AD mice. It was characterized by drastically reduced general activity, as measured in terms of time, distance covered, and rearing in the walls. Low performance, with long-lasting periods of freezing in the center of the field, resulted in an inverted pattern for thigmotaxis. Walking speed patterns were also found disrupted. Emotionality, as measured by defecation and urination, was increased.

Spontaneous animal models with face validity for OCD, such as the “BIG mice” (Greene-Schloesser et al., 2011; Mitra et al., 2017) are based on their divergent nesting and marble-burying behaviors, increased compulsive-like digging, with respect to “SMALL mice” counterparts that exhibit risk assessment and inhibition of movement in the open-field and the elevated plus-maze. These animals also diverge in the thigmotaxis, and the rearing frequency in the periphery is three-fold higher in the “BIG mice” compared to the “SMALL.” In the present work in 3xTg-AD mice, the temporal course and intensity of digging behavior in the marble test were found increased in the isolated mice. Here, the marble-test experimental setup was similar to the one used in the corner test except for marble pieces and the starting position facing the wall. As compared to other laboratory protocols, the number of marble pieces was reduced to 12, and they were allocated in one half of the test cage to reduce the chance that differences in buried pieces be due to differences in locomotor activity (Nicolas et al., 2006; Thomas et al., 2009). Still, as shown in the open-field test, the NTg mice were the active animals, similar to “BIG mice,” and both 3xTg-AD mice subgroups exhibited the same anxiety/fear-like behavior. Therefore, the increased number of pieces buried in isolated 3xTg-AD mice could not result from locomotor activity since this was drastically reduced, whereas it was normal in NTg mice. Both subgroups of 3xTg-AD mice showed similar anxiety levels but differed in the corner and marble tests' digging signatures. In this regard, some pharmacological studies have noted that while the dose-dependent reduction in marble-burying could be due to several drugs, this effect was not always related to their locomotor effects (Jimenez-Gomez et al., 2011). Due to this controversy, the open-field or other anxiety tests that also monitor the general activity are a must for interpretations and discard confounding factors.

The genetic background is determinant for the expression of behaviors (Albelda and Joel, 2012). Also, animal models based on overexpression of human transgenes from familial forms of the disease are not exempted from limitations and shortcomings. The main translational gap, but also a challenge, is the difficulty to fully recapitulate the complexity of neurological and psychiatric patterns observed at the clinical level in the human patient (Giménez-Llort et al., 2006, 2007; Kosel et al., 2020). Other critical issues refer to genetic engineering since transgene insertions can have phenotypic consequences and could confound some experiments (Goodwin et al., 2019). The present results used the new Spanish colonies of 3xTg-AD mice established at Universitat Autònoma de Barcelona after embryonic transfer on a congenic C57BL/6J background from progenitors with a hybrid C57BL/6J x 129 Sv genetic background kindly provided by Prof. Frank M LaFerla in 2011. This animal model, also provided by The Jackson Lab as Stock #004807, is adapting this genetic strategy, with animals on congenic C57BL/6J or 129S4 expected to be available beginning last January 2019^2^ Concerning our work, we can compare the present results with those already published in our laboratory using the first colonies of NTg and 3xTg-AD mice in the C57BL/6J x 129 Sv genetic background. Those results showed that, at this old age, 3xTg-AD mice's performance was already disrupted in the marble test (Torres-Lista et al., 2015). Their pattern was similar to that exhibited here by 3xTg-AD mice that lost their partners. Thus, it seems that in the present work, the C57BL/6J genetic background induces a delay in the expression of the disruption, but the pattern emerges in the animals that have confronted social isolation in the last 2 months. This would agree with the extended window of observation in the corner test, allowing to detect of reduced rearing in both subgroups of 3xTg-AD mice, with different degrees of statistical significance (worse in isolated). Furthermore, we recently reported that in the social interaction test (Torres-Lista and Giménez-Llort, 2019), the ethogram of 14-month-old male and female 3xTg-AD mice points at digging as a robust and consistent non-social interaction behavior. In that context, digging emerged on minute three of the test together with self-grooming. In females, the behavior was elicited earlier in the ethogram (on average, at 90 s), in agreement with their increased anxious-like profile and shrinking the action program described by Lát (1973). Thus, although the repetitive and compulsive behaviors that characterize the OCD are defined as “meaningless,” they are also considered to decrease the resulting distress.

In other animal models of AD such as the APP/PS1, Tg2576, and 5xFAD mice, several authors had reported a social isolation-induced increase in amyloid pathology (Hsiao et al., 2011; Huang et al., 2011; Peterman et al., 2020). In contrast, in 3xTg-AD mice, limited impact was found when long-term isolation was implemented from postweaning to adulthood (Pietropaolo et al., 2009). However, in our most recent work with these new Spanish colonies of 3xTg-AD mice, and also studying naturally occurring long-term isolation due to loss of partners in an aged scenario, we found that tau pathology of 3xTg-AD mice was enhanced, albeit did not reach the statistical significance, probably due to a celing effect (Muntsant and Giménez-Llort, 2020a). Nevertheless, the hippocampal atrophy asymmetry found in human patients with AD (Wachinger et al., 2016) was modeled for the first time in mice and allowed us to show its worsening after long-term isolation. Further analysis has also found brain differences in the cortical areas (Muntsant and Giménez-Llort, 2020b). In the present work, the effects of a short-isolation did not modify the general motor activity pattern nor the anxiety-like profile of 3xTg-AD mice. However, as we have shown recently (Muntsant and Giménez-Llort, 2020a), a longer isolation regime induced re-structured negative valence system with the emergence of bizarre behaviors and flight copying-with-stress strategies were found. More importantly, with regards of the present work, the long-term period induced a prominent hyperactive pattern in both gross (general motor activity in most tests) and fine-motor functions (increased nesting-behavior) that were correlated to hippocampal tau pathology (Muntsant and Giménez-Llort, 2020a). Activation of cortical and striatal regions during the expression of a naturalistic compulsive-like behavior in the rabbit have been described (Cano-Ramírez and Hoffman, 2018), so these are also our target areas in our current investigations. Excessive nest building has been described as a unique behavioral phenotype in the deer mouse model of obsessive-compulsive disorder (Wolmarans et al., 2016). As mentioned before, psychogenetic bidirectional selection of mice for nesting behavior is used as an animal model for compulsive-like behaviors, and it is also leading to distinct marble-burying (Greene-Schloesser et al., 2011; Mitra et al., 2017).

Thus, despite the current brief report could be considered preliminary, our precedent data describing the consistency of digging in males studied in a social paradigm (Torres-Lista and Giménez-Llort, 2019) and of increased nest-building in animals under a long-term isolation (Muntsant and Giménez-Llort, 2020a) support the consistent observations during housing routines that lead to this work and the present results showing disruptions in the subgroup under isolation. Still, the limitations of the current work must be noted and are mainly due to the naturalistic scenario, since the natural death of home-cage partners at this age only affects to 3xTg-AD mice (Giménez-Llort et al., 2008). This fact also limits the sample size of the animals under a “naturalistic isolation.” To solve it, in the ongoing investigations, a forced isolation paradigm is being used for both genotypes and including both sexes. Studying digging signatures in other paradigms and the impact of isolation at different ages/stages of disease is also a matter of current investigations (Marin-Pardo and Gimenez-Llort, 2020).

Among our different reports on non-pharmacological preventive/therapeutic interventions in this mouse model, handling was able to modify marble-burying (Torres-Lista et al., 2015) and the long-lasting effects of early-life interventions (postnatal handling and environmental enrichment) were also observable in other perseverative behaviors (Torres-Lista and Giménez-Llort, 2015). However, in another model, the 5xTg-AD mice, Petermam et al. showed that prolonged isolation stress accelerated the onset of disease-related pathology despite running wheels and environmental enrichment (Peterman et al., 2020). Still, at the clinical level, the beneficial effects of these interventions foreseen in the context of the pandemic are supported by the literature (Davim et al., 2020). Our preliminary results, with regards to the implementation of a stimulation program to improve both psychological and motor functions in a long-term care center, are encouraging (Castillo-Mariqueo et al., 2020), but also indicate that the time frames for intervention seem to be critical and depend on the frailty of aging/AD scenarios (Giménez-Llort, 2010).

In summary, as a translational neuroscience approach, the present brief report in the 3xTg-AD mice can be useful to estimate the impact of a short-isolation regime with regards to the emergence or enhancement of compulsive behaviors in Alzheimer's disease where NPS/BPSD-like symptomatology is already present. The prevalence and incidence, the temporal course, and the intensity of this behavior were found to increase in the 3xTg-AD mice that had lost their “room partner” despite having lived in social structures for more than 10 months, since they were born. When they were tested under neophobia conditions, the incidence of this behavior was smaller (delayed), and the pattern of digging was disrupted, resembling that previously reported for worse stages of the disease. Despite the limitations of a naturalistic isolation (small sample and only affecting one genotype), together with precedent results of long-term isolation enhancing nesting-behavior, we present this combined paradigm unveiling distinct features of digging signatures as it can be useful to study these perseverative behaviors, their complex interplay with anxiety states already present in the AD scenarios and its worsening by naturalistic or forced isolation.

## Data Availability Statement

The raw data supporting the conclusions of this article will be made available by the authors, without undue reservation.

## Ethics Statement

The animal study was reviewed and approved by CEEAH Universitat Autònoma de Barcelona and DMAH Generalitat de Catalunya.

## Author Contributions

LG-L: concept and draft manuscript. Both authors equally contributed to the performance and analysis of the experiments. Both authors revised and approved the final manuscript.

## Conflict of Interest

The authors declare that the research was conducted in the absence of any commercial or financial relationships that could be construed as a potential conflict of interest.

## Footnotes

^1^APA Obsessive-compulsive disorder https://www.psychiatry.org/patients-families/ocd/what-is-obsessive-compulsive-disorder.

^2^Alzforum.org (2020) https://www.alzforum.org/research-models/3xtg.
